# Living with mental health issues: citizen science project on self-management strategies

**DOI:** 10.1038/s44184-025-00166-2

**Published:** 2025-10-11

**Authors:** Mike Slade, Olamide Todowede, Doreen Boyd, Colleen Ewart, Akemi Hara, Fred Higton, Stuart Moran, Julie Repper, Dan Robotham, Emily Slade, Angela Sweeney, Stefan Rennick-Egglestone

**Affiliations:** 1 School of Health Sciences, Institute of Mental Health, University of Nottingham, Nottingham, UK; 2 Nord University, Faculty of Nursing and Health Sciences, Health and Community Participation Division, Namsos, Norway; 3 West Midlands Evidence Synthesis Group, Centre for Evidence and Implementation Science, School of Social Policy and Society, University of Birmingham, Birmingham, UK; 4 School of Geography, University of Nottingham, Nottingham, UK; 5 Recovery Research Team Lived Experience Advisory Panel, Nottingham, UK; 6 Medical Governance Research Institute, Tokyo, Japan; 7 Nottingham BRC Mental Health Involvement Team, Nottingham, UK; 8 Information Services, University of Nottingham, Nottingham, UK; 9 ImROC, Nottingham, UK; 10 Wellcome Trust, London, UK; 11 King’s College London Service User Research Enterprise (SURE), London, UK; 12 National Institute for Health Research (NIHR) Nottingham Biomedical Research Centre, Institute of Mental Health, Nottingham, UK

**Keywords:** Patient education, Outcomes research, Rehabilitation

## Abstract

People living with mental health issues use a range of self-management strategies. Most strategy recommendations have been developed by clinicians and researchers, so they may not reflect the full range of approaches used in practice. A citizen mental health science methodology can address this bias in strategy identification. We co-created a list of 77 pre-defined self-management strategies, and 1116 public contributors (*n* = 468 mental health service users, *n* = 497 lived experience not using services, *n* = 151 no lived experience) living in the United Kingdom completed an online survey identifying their use of each strategy, and identifying extra strategies. A wide range of pre-defined strategies were used by contributors, with differences in usage patterns identified between the three groups. 401 distinct extra strategies were identified. The active use of avoidance as a self-management strategy was more common than anticipated, including avoiding alcohol, social media, thinking about problems, other people, and mental health services.

## Introduction

Self-management is an important approach to mitigating the impact of long-term disorders. Self-management enables people with ongoing conditions to “*make informed choices, adopt a new perspective and generic skills that can be applied to new problems as they arise, to practice new health behaviours and to maintain or regain emotional stability*”^1^. Some definitions of self-management focus on behavioural aspects, such as behaviours to control symptoms, prevent relapses, and optimise health”^2^. Some reviews restrict the meaning of ‘self-management’ to clinically-provided interventions, such as self-management training^3^. However, in this article, we apply a broader lens to encompass also (a) choosing to use an optional mental health intervention (e.g., attending a Recovery College) and (b) the intentional use of new lifestyle behaviours (e.g., joining a fitness class) and cognitive strategies (e.g., cultivating mindfulness) initiated by the individual.

Self-management has been measured as the concept of ‘patient activation’, which involves knowledge, skills, and confidence to manage one’s health^4^. Higher patient activation is associated with better patient outcomes^5^, such as more favourable health-related behaviours and improved quality of life^6^. It is also associated with lower use of services^7^, for example, reduced general practice and emergency department use^8^.

Self-management is relevant to mental health^9^. A study of self-management strategies used by people (*n* = 50) living with mood and anxiety disorders identified five categories: social; existential; functional; physical; and clinical^2^. Examples of specific approaches (e.g., finding meaning for Existential; creating a routine for Functional) are given. A systematic review (37 trials, 5790 participants) found self-management interventions as a whole for people with severe mental illness improved outcomes of symptomatology, functioning, quality of life, recovery, hope, empowerment, and self-efficacy^10^. Four categories of intervention were identified: psycho-education, relapse prevention, coping skills, and medication management. The impact of each category or of individual interventions on outcome was not investigated in this or other systematic reviews^3,11^, with all approaches pooled as self-management interventions. Beyond mental health, one systematic review focusing on a population of adults with chronic physical diseases who are experiencing depressive symptomatology (15 studies, 2064 participants) did consider types and elements of self-management intervention^12^. Their moderator analysis found no impact on outcome for intervention length, duration, or self-directed versus clinician-delivered. The self-management strategies of ‘decision-making’ and ‘taking action’ moderated the outcome.

People with severe mental illness may also benefit in relation to physical health outcomes^3^. Diagnostic groups may differ: higher patient activation was found in people with bipolar disorder than major depressive disorder, and lower patient activation in people with schizophrenia^13^. Similarly, diverse self-management strategies are used synergistically by people with mood and anxiety disorders to reduce symptomatology and support recovery^2^. Online self-management strategies are a potential growth area for mental health support^14^, including interventions targeting psycho-education, medication management, communication and shared decision-making, management of daily functioning, and experience sampling monitoring^15^.

Self-management strategies are intended to promote clinical recovery, including reduced symptomatology, improved functioning, and better relapse prevention. Strategies to meet this aim have two key features. First, they are developed by mental health workers and researchers rather than by people living with mental health issues. The knowledge, behaviour, and other changes they target consequently reflect clinical priorities. Second, while consideration is given to implementation influences such as patient preference, strategies are primarily recommended by clinicians in relation to diagnostic groups.

An international consensus has emerged about orienting mental health systems towards personal recovery^16^. This re-orientation places a stronger emphasis on the lived experience of individuals, and involves personalised rather than diagnostically-driven approaches to supporting recovery. A focus on personal recovery challenges assumptions that have underpinned the development of self-management interventions.

First, more emphasis is needed on learning from the ways people live with mental health issues, if the ecological validity of recommendations is to be maximised. A recovery approach recognises that people with mental health issues have much more in common with people without mental health issues than differences^17^. So rather than needing distinct treatments or interventions, many life challenges experienced by people with mental health issues are everyday difficulties, which need mainstream rather than specialised solutions^18^. This was investigated in a study asking staff about wellbeing influences for themselves and for their service users^19^. Responses relating to personal well-being were primarily strengths-oriented, e.g. the importance of achieving balance between social roles (work, family, etc.), striving for high ambition goals, and the increased wisdom that comes with ageing. This contrasted with the primarily deficit-oriented view of what their service users needed, e.g,. The importance of balancing the negative illness-related impacts, setting low-ambition goals (‘therapeutic realism’), and the benefit of lowered expectations through improved insight, which comes with ageing. Clinician-driven self-management recommendations may be influenced by non-valid assumptions arising from in-system stigma^20^, and hence may lack ecological validity.

Second, individuals live well in multiple ways, not all aligned with optimising health. Recovery involves more than high treatment adherence and healthy lifestyles; it involves diverse approaches to experiencing connectedness with self, others, and the world, improving hope, developing a positive identity, finding a personally valid meaning to experiences, and empowerment to make choices about oneself and one’s life^21^. Neither diagnosis nor clinical recommendation is sufficient to identify how someone should live their life^22^. An implication of the human right to self-determination is that finding how to live well is always a personal journey.

To broaden the lens, we use the term self-management to encompass both the clinical meaning of interventions to support improvement in health outcomes, such as symptomatology, and the everyday meaning of how people either actively (doing something) or by omission (not doing something) live with mental health issues, support their own recovery, and look after their own wellbeing. Clinical and everyday strategies will differ. Clinical recommendations do not support many widely-used strategies, e.g,. using alcohol for stress relief, avoidance as a strategy for problem management, or discontinuing healthcare as an act of agency.

Identifying the full range of self-management strategies people actually use will bring many benefits: making visible the widest choice of strategies; providing a source of innovation for new clinical interventions; allowing formal evaluation of the benefits and harms arising from different strategies; supporting people in managing possible harms arising from clinically not recommended strategies, e.g. people using ketamine to manage treatment-resistant depression would benefit from purity testing kits, and people self-medicating with alcohol would benefit from rapid scans to detect early-stage liver fibrosis^23^; and contributing to the broader movement towards centring clinical care on lived experience rather than professional expertise.

Methodological innovation is needed to move beyond the clinical assumptions that permeate researcher-led studies. An innovative approach to knowledge creation is citizen science^24^, defined as public engagement in scientific research activities, where citizens actively contribute to science either with their intellectual effort, surrounding knowledge, or their tools and resources^25^. The 10 principles for citizen science, proposed by the European Citizen Science Association^26^, are widely accepted, and include: projects actively involved citizens in a scientific endeavour that generates new knowledge or understanding; projects have a genuine science outcome; both professional scientists and citizen scientists benefit from taking part; and citizen scientists may if they wish participate in multiple stages of the scientific process. Citizen science is now beginning to be used in mental health research^27^, and has the potential to provide a counter-balance for clinician and researcher assumptions.

This project aimed to use citizen science to understand the full range of self-management approaches used by people to manage their own mental health problems.

## Methods

This project was conducted as part of the Citizen Science To Achieve Co-production At Scale (C-STACS) programme. Two types of citizen mental health science have been distinguished: contributory citizen science is predominantly researcher-initiated, with many citizens making specific contributions such as data collection, and co-created citizen science involves citizens and researchers jointly designing and conducting studies to support the democratisation of science^28^. C-STACS incorporates both approaches. It was conducted in collaboration with 12 representative stakeholders from 11 organisations chosen for diverse perspectives on the mental health care system: Centre for Mental Health, ImRoC, McPin Foundation, Mental Elf, National Health Service (NHS) Confederation, National Survivor User Network (NSUN), National Institute for Health and Care Research (NIHR) Nottingham Biomedical Research Centre (two Patient and Public Involvement representatives), Nottinghamshire Healthcare NHS Foundation Trust, Service User Research Enterprise (SURE), Social Spider, and Taraki. These representative stakeholders who constituted the C-STACS Advisory Board met regularly throughout the study. C-STACS was led by five researchers, with input from the Advisory Board at every stage from design to dissemination. Individuals with mental health lived experience were involved at all stages, including in the study design, as co-applicants at the funding acquisition stage, as research project leads, as C-STACS Advisory Board and Lived Experience Advisory Panel (LEAP) members, and as co-authors on this manuscript. Regarding terminology, citizen science uses the term ‘projects’ for studies and ‘contributors’ for participants. Ethical approval was obtained (University of Nottingham FMHS 349–0723, approved 22/08/23) and all participants gave online informed consent.

### Procedures

The Advisory Board and researchers co-created a preliminary list of mental health self-management approaches, based on reviews of clinically recommended self-management strategies^10,29–31^. The preliminary list was reviewed by the Recovery Research Team Lived Experience Advisory Panel (LEAP), comprising 10 individuals with lived experience of mental health issues and expertise in Patient and Public Involvement, who added items from personal experiences. After refinement following this review, the list was commented on by the C-STACS Advisory Board, to increase comprehensiveness and cross-cultural relevance. The final list of 77 self-management strategies was developed by the researchers based on these comments. We then developed and piloted an online survey (Supplementary material 1) on Microsoft Forms to collect information from citizens about their use of these self-management strategies. Strategies were thematically grouped into superordinate categories, with those considered from a clinical perspective to either raise ethical or legal issues, or to have potential for harm to self or others, also categorised as ‘not recommended’. Categories comprised: Accessing support from the mental health system (*n* = 12); Individual approaches (clinically recommended) (*n* = 44); Individual approaches (not recommended) (*n* = 4); Social approaches (*n* = 14); and Spiritual engagement (*n* = 3).

### Recruitment and survey data collection

We invited contributors to take part in the project by completing our form. Inclusion criteria for public contributors were: aged over 18, and living in the United Kingdom. Personal experience of mental health problems was not a formal inclusion criterion, to enable contributions from people who would not normally self-describe as having mental health problems, but who may still be using self-management strategies. Advertising materials described the project as investigating how individuals manage their mental health.

The survey was publicised online. An organisation specialising in the use of social media to recruit health research participants created a web-based landing page, and spent project funds boosting the reach of social media messages, drawing attention to this landing page. They adaptively moved spending between posts on major social media platforms, to maximise return on investment. The 11 stakeholder organisations distributed information publicising the landing page by email and through posts on social media websites. The project team posted information onto existing citizen science mailing lists that are routinely used to promote citizen science projects.

All procedures took place online, and data were collected between December 2023 and September 2024. After giving consent and completing demographic details, contributors rated each pre-defined self-management strategy using a 5-point Likert scale describing the frequency of personal use (Never, Rarely, Sometimes, Very often, Always). Contributors were then asked to identify in a text box any further mental health self-management strategies they use that were not in the pre-defined list. After completing the survey, participants were given an option to join our mental health citizen science community mailing list, including to learn about project findings and new mental health citizen science projects. An opportunity to be involved in analysis was offered to mailing list members and through social media. The message stated, ‘We are just beginning the data analysis for the citizen science project you participated in, as we are currently familiarising ourselves with the content. If you are interested in assisting in putting contextual meaning to the data analysis or the reporting, we encourage you to indicate your interest <here>. Your involvement could further enrich the insights gained from the information generated from this project. No public contributors took up the offer.

### Analysis

Missing quantitative data (1596 (2%) of 85,932 data points) on strategy use were re-coded as Never used. Likert scales were converted to a quantitative score from 0 (Never) to 4 (Always). We aggregated participant ratings for all 77 strategies into a single combined set of values, and derived the overall median and interquartile range (IQR) to assess the central tendency and variability of strategy use across the entire sample. Then we computed the median (IQR) rating for all strategies and the median (IQR) for these 77 median values to assess the central tendency and spread of strategy use. For each strategy, we then computed the median (IQR) for all participants and then separately within each of the three experience groups (No mental health lived experience, Lived experience without service use, Mental health service user), before evaluating between-experience group differences using the Kruskal-Wallis test. As a sensitivity analysis, both False Discovery Rate (FDR) and Bonferroni correction (significance threshold 0.05/77) were calculated. Ordinal logistic regression models were used to investigate usage predictors, using experience group, self-reported Gender (Male, Female), Education (Primary/Secondary, Degree/Higher degree), Ethnicity (White British, Other), Age (six bands), and Income (six bands) as independent variables.

The extra strategies were prepared for analysis by anonymising contributor-identifying information, deleting non-strategies (e.g., concerns about mental health care, strategies to avoid, comments on current life situation), grouping near-identical strategies, and deleting strategies already contained in the list of 77 predefined strategies (whilst retaining candidate refinements to the predefined strategies, for incorporation into the integrated list). The remaining strategies were then inductively thematically organised into emergent themes, through iterative consultation between analysts to generate meaningful grouping,s including separation of clinically recommended versus not recommended strategies. Finally, the predefined and extra strategies were integrated into one structured list.

## Results

The characteristics of the 1116 public contributors are shown in Table 1.

**Table 1:** Characteristics of project contributors (*N* = 1116)

	*n* (%)
**Ethnicity**	
White British	923 (83)
European	42 (4)
Asian/Asian British	35 (3)
White Irish	24 (2)
Black African/Caribbean / British	21 (2)
Mixed	18 (2)
Arab	4 (0)
Gypsy, Roma or Irish Traveller	4 (0)
Latin	4 (0)
Not known/prefer not to say	41 (4)
**Self-reported gender**	
Female	874 (78)
Male	212 (19)
Non-binary	18 (2)
Prefer not to say	12 (1)
**Age**	
18–24	151 (14)
25–34	103 (9)
35–44	162 (15)
45–54	255 (23)
55–64	351 (31)
65+	94 (8)
**Education**	
Primary/Early years	12 (1)
Secondary	383 (34)
Degree	329 (29)
Higher degree	306 (27)
Prefer not to say	86 (8)
**Experience**	
Mental health lived experience	965 (86)
Mental health service user	468 (48)
Not a mental health service user	497 (52)
Informal carer	119 (11)
Mental health worker	64 (6)
No mental health experience	95 (9)
**Income**	
Less than £10,000	285 (26)
£10,000-£24,999	316 (28)
£25,000-£49,999	220 (20)
£50,000-£74,999	69 (6)
£75,000-£99,999	26 (2)
£100,000+	18 (2)
Prefer not to say	182 (16)

### Predefined strategies

The use of the 77 predefined strategies by the public contributors was investigated.

Ratings for the 77 predefined strategies were Never (41,737, 49%), Rarely (10,961, 13%), Sometimes (15,402, 18%), Very often (9481, 11%) and Always (8351, 10%). The median (IQR) rating of strategy use across the 1116 participants (i.e., how much the average participant makes use of each strategy) was 1 (0,2), corresponding to Rarely. The median (IQR) rating of strategy use across the 77 strategies (i.e., how much the average strategy is used) was also 1 (0,2). In other words, most of the 77 pre-defined strategies were occasionally used by the public contributors.

The frequency of use of all predefined strategies is shown in Supplementary material 2, along with comparison of use between experience groups. The 24 strategies with a median rating of at least 2 (Sometimes used) are shown in Table 2.

**Table 2:** Frequently-used self-management strategies (*n* = 24)

	Frequency *Sometimes (2), Very often (3) or Always (4)*				Difference Unadjusted *p*-value
**Contributors**	**All**	**No lived experience**	**Lived experience, no service use**	**Mental health service user**	
*n*	1116	151	497	468	
**Category: accessing support from the mental health system**	Median (IQR)				
I choose to take my medication as prescribed	3 (0,4)	0 (0,4)	1 (0,4)	4 (1,4)	<0·01
**Category: individual approaches**					
Listening to music	3 (2,4)	3 (2,4)	3 (2,4)	3 (2,4)	0·81
Watching TV	3 (2,4)	3 (2,3)	3 (2,4)	3 (2,4)	0·02
Distracting myself	3 (2,4)	2 (1,3)	3 (2,4)	3 (2,4)	<0·01
Avoiding situations or people that trigger my distress	3 (2,3·25)	2 (1,3)	3 (2,3)	3 (2,4)	<0·01
Have a cup of tea	3 (1,4)	3 (0,4)	2 (1,4)	3 (1,4)	0·17
I spend time with nature, e.g. in parks, with animals	2 (2,3)	3 (1,3)	2 (1·25,4)	2 (2,3)	0·83
Take a bath or shower	2 (2,3)	3 (2,4)	2 (2,3)	2 (1,3)	0·02
I engage in exercise, e.g., walk/jog/run/cycle	2 (1,4)	3 (2,4)	2·5 (1,4)	2 (1,3)	0·03
Play with a pet	2 (0,4)	2 (0,4)	2 (0,4)	2 (0,4)	0·51
Participate in physical activity	2 (1,3)	2 (1,4)	2 (1,3)	2 (1,3)	0·05
Going to sleep early or changing my sleep patterns	2 (1,3)	2 (0,2)	2 (1,3)	2 (1,3)	0·01
Doing household chores, e.g., tidying up, washing clothes	2 (1,3)	2 (1,3)	2 (1,3)	2 (1,3)	0·69
Daydreaming	2 (1,3)	2 (1,3)	2 (1,3)	2 (1,3)	0·26
Reading	2 (1,3)	2 (1,3)	2 (1,3)	2 (1,3)	0·77
Mindfulness	2 (0,3)	2 (0,3)	2 (0,3)	2 (0,3)	0·04
Activities to focus/reset on the nervous system, e.g., stretching, colouring, gaming	2 (0,3)	1 (0,2)	2 (0,3)	2 (1,3)	<0·01
Counting my blessings	2 (0,3)	2 (0,3)	2 (0,3)	2 (0,3)	0·36
Keeping busy at work	2 (0,3)	2 (0,3)	2 (0,3)	2 (0,3)	0·18
Cook or bake your favourite foods	2 (0,2)	2 (0·5,3)	2 (0,2)	2 (0,2)	<0·01
**Category: social approaches**					
Use social media	3 (2,4)	3 (2,4)	2 (1,3)	3 (2,4)	<0·01
Enjoying quality family time	2 (1,3)	3 (2,4)	2 (1,3)	2 (1,3)	<0·01
Being with support systems, e.g., trusted friends, families	2 (1,3)	2 (1,3)	2 (1,3)	2 (2,3)	0·05
Meet with friends	2 (1,3)	2 (1,4)	2 (1,3)	2 (1,3)	<0·01

Overall, 47 (61%) of the 77 self-management strategies showed statistically significant differences in usage between experience groups using unadjusted *p*-values. The same pattern remained in attenuated form after adjusting using FDR (39 significant differences) and Bonferroni (21 significant differences). The overall pattern of self-management strategy use differing between the three groups – people with no lived experience, with lived experience and no service use and current service user – is robust. Mental health service users were, in comparison to contributors with no lived experience, more likely to choose to take medication as prescribed, use distraction or avoidance, or undertake activities to focus or reset the nervous system, such as colouring or stretching.

The median (IQR) for Accessing support from the mental health system (12 aggregated strategies) was 0 (0,1), for Individual approaches (clinically recommended) (44 strategies) was 1 (0,2), for Individual approaches (not recommended) (4 strategies) was 0 (0,2), for Social approaches (14 strategies) was 1 (0,3), and for Spiritual engagement (3 strategies) was 0 (0,0). None of the five categories of self-management strategy were markedly more used than the other categories.

Usage predictors of the seven strategies used Very often or more were investigated (Supplementary material 3). *I choose to take my medication as prescribed* was used more by mental health service users, and by people of older age and with lower levels of formal education. *Listening to music* was used more by younger individuals. *Watching TV* was used more by females, people with lower education, and White British individuals. *Distracting myself* was used more by both lived experience groups and by females. *Avoiding situations or people that trigger my distress* was used more by mental health service users and people of older age. *Have a cup of tea* was used more by females and older age. Finally, *Using social media* was used more by mental health service users, females, and by younger contributors and those with lower formal education.

### Extra strategies identified by public contributors

The types of extra strategies, beyond the 77 predefined strategies, were investigated.

In relation to the survey text-box for extra strategies, 623 (56%) of the 1116 public contributors did not respond and a further 58 (5%) said None or Not applicable or equivalent, leaving 435 (39%) with a valid response. Of these, 293 contributors identified one strategy, 82 identified two strategies, 29 identified three, 10 identified four, 9 identified five, and 12 identified 6–13 strategies. A total of 729 extra strategies were identified. After grouping near-identical strategies, 497 distinct strategies were provided. After deleting strategies already listed in the survey, 401 new distinct strategies remained. These were thematically analysed to produce a final coding framework identifying a further 31 strategies (22 clinically recommended, 9 not recommended) not included in the list of predefined strategies. The coding framework for these strategies is shown in Table 3.

**Table 3:** Coding framework for extra strategies (*n* = 31)

Theme	Definition	Examples	Strategies within theme (*N* = 401) *n* (%)
**1 Cognitive strategies (clinically recommended)**	**Using thinking strategies**		**34 (8)**
1.1 Positive thinking	Using positive thinking strategies	Positive affirmations. Self-positivity. Reflective practice.	15 (4)
1.2 Acceptance and resilience	Focussing on acceptance and building resilience	Accepting that we are what we are. Knowing that I’ve felt bad before and it will pass. Understanding that low mood and stresses are natural occurrences of life.	19 (5)
**2 Cognitive strategies (not recommended)**	**Using thinking strategies that are not clinically recommended**		**10 (2)**
2.1 Avoid thinking about problems	Actively avoiding thinking about problems	Avoid thinking about it. Dissociation. Compartmentalisation.	10 (2)
**3 Behavioural strategies (clinically recommended)**	**Using behavioural strategies**		**139 (35)**
3.1 Nature	Engaging with nature	Cold, wild, and open water swimming. Forest bathing. Watching a thunderstorm. Planting a tree.	12 (3)
3.2 Travel	Travelling	Going on holiday. Driving places. Spur-of-the-moment activities. Moving area. Walking along the beach. Going to festivals. Going to the cinema. Horse racing.	28 (7)
3.3 Work	Using employment and occupation as a self-management resource	Retirement. Talking to my manager. Going to work.	5 (1)
Leisure	Undertaking leisure activities	Games. Relaxing. Audiobooks and books. Creative activities. Music. Masturbation. Hobbies.	65 (16)
3.5 Living space improvements	Improving my home space	Rearranging my living space. Tidying my space. Decluttering. Decorating.	10 (2)
Lists and planning	Using strategies to make future plans	Writing lists of daily tasks. Sorting your finances. Planning for the future. Setting alarms to remember to eat and drink.	16 (4)
3.7 Healthy eating	Eating a healthier diet	Healthy eating. Diet. Carnivore diet.	3 (1)
**4. Behavioural strategies (not recommended)**	**Using behavioural strategies that are not clinically recommended**		**23 (6)**
4.1 Deliberate self harm	Harming myself to manage my feelings	Self harm. Suicide attempts. Cutting. Planning to end my life. Starving myself. Taking risks.	10 (2)
4.2 Overeating	Comfort eating	Overeating. Comfort eating. Eating junk food. I eat buns, chocolate, crisps. Binge eating.	5 (1)
4.3 Under- or over-sleeping	Changing how I sleep	Naps. Sleeping for a long time. Not sleeping.	3 (1)
4.4 Non-prescribed medications	Using non-prescribed drugs	Weed. Psilocybin. Sleeping tablets. Antidepressants without a prescription.	5 (1)
**5. Avoidance strategies (clinically recommended)**	**Using avoidance strategies**		**9 (2)**
5.1 Avoid alcohol	Reducing my alcohol consumption	Keep drinking to the weekend. Never touch alcohol.	2 (0)
5.2 Avoid social media or devices	Reducing social media and internet use	Social media timeouts. Mute people on social media. Not answering emails. Turning off devices. Significantly reducing internet, social media and TV use.	7 (2)
**6. Avoidance strategies (not recommended)**	**Using avoidance strategies that are not clinically recommended**		**18 (4)**
6.1 Isolate	Isolate myself away from other people	Isolating. Staying in bed. Laying in bed. Keeping to myself. Being alone. Disappearing. No romantic relationships.	16 (4)
Avoid mental health services	Avoid contact with mental health services	Avoid mental health professionals. Avoid statutory mental health services.	2 (0)
**7. Affective strategies (clinically recommended)**	**Using emotional strategies**		**85 (21)**
7.1 Crying	Actively using crying to manage my feelings	Crying. Crying myself to sleep. Crying in the shower. Screaming into the void.	6 (1)
7.2 Laughing	Actively using laughing to manage my feelings	Watching comedy. Humour. Laughing. Telling jokes.	5 (1)
7.3 Gratitude	Cultivating a grateful attitude	Practising gratitude.	3 (1)
7.4 Emotional self-regulation	Using specific strategies to manage my feelings	TDCS. Delta wave music. Worry worms. Dot-to-dot books. Tapping. Fidget toys. Stimming. Cold showers. Looking through photos of happy times. Standing barefoot on wet grass. Shaolin practice. Vagus nerve stimulation. Breathing techniques.	41 (10)
7.5 Mental health apps	Using mental health apps	Named apps: Calm, Stay Alive, Loona, Wysa, Happify, Aura, Headspace.	9 (2)
7.6 Self care	Pampering myself with treats and experiences	Shopping. Getting a massage. Weighted blanket. Painting my nails. Having a lie in. Change my appearance like dyeing/cutting my hair. Ordering food when I don’t feel able to cook.	21 (5)
**8. Affective strategies (not recommended)**	**Using emotional strategies that are not clinically recommended**		**20 (5)**
Complementary therapies	Using complementary therapies	Butterfly hug EMDR. Cryotherapy. Acupuncture. Reiki. Hypnotherapy. Aromatherapy. Medical cannabis. Lion’s Mane. Vitamins. CBD oil.	15 (4)
8.2 Complain and argue	Having a cathartic argument	Making complaints. I annoy my partner. Bitching and complaining with other mad people.	5 (1)
**9. Social strategies (clinically recommended)**	**Using social strategies**		**43 (11)**
Help others	Helping other people	Helping others. Compassion for others. Try to do an act of kindness. I help those I know locally who are vulnerable. Teaching about mental health.	14 (3)
Animals	Engaging with animals	Dog walking. Horse riding. Butterfly watching. Staring at my fish tank.	8
Socialise	Doing social activities	Crochet club. Writing groups. Talking. Phoning a friend. Talking to my dog about my problems. Seeing my family thrive.	21
**10. Educational strategies (clinically recommended)**	**Using educational strategies**		**20 (5)**
10.1 Learn about mental health	Using educational resources to learn about mental health self-management	Learning new skills to avoid dissociation. Psychoeducation. Self-help books. Stress management course. Understanding how traumas work.	11
10.2 Adult education	Learning and lifelong development, especially in areas beyond mental health	Internet research. Degree or short courses. Learning about other cultures. Genealogy research. Gaining a psychology degree so I can advocate for myself.	9

## Discussion

A wide range of self-management strategies identified in previous research were found to be used, but with different usage patterns between people with versus without mental health lived experience, and between those using versus not using mental health services. In particular, service users more frequently used prescribed medication, cognitive and behavioural avoidance, and social media. Diverse extra strategies were identified, including nine that would not be clinically recommended due to potential for harm.

The extra strategies were characterised by three features. First, avoidance was an actively used strategy, for example, avoiding alcohol use, stopping social media, not thinking about problems, isolating from other people, and, most challengingly, disengaging from mental health services. While avoidant coping is routinely viewed by clinicians as maladaptive and hindering of problem-solving and resilience, more nuanced theoretical understandings of the functional benefits of avoidance are emerging, such as adaptive avoidance (e.g., distancing), avoidance coping (e.g., by disengagement) and proactive avoidance (e.g., leaving a toxic social network)^32^. Future research might focus on influences on strategy choice, such as perceived or actual availability of more active strategies to people who live with long-term or disabling mental health problems.

Second, public contributors identified mental health-related learning and wider adult education as important strategies. Clinician signposting to relevant apps, podcasts, and online / local in-person educational opportunities beyond Recovery Colleges^33,34^ is indicated.

Third, active use of a wide range of emotional self-regulation approaches was identified, including behavioural changes in diet, sleep architecture, and self-harm behaviour. These behaviours might be understood diagnostically as symptoms, whereas public contributors identified them as self-management strategies. The citizen mental health science methodology was intended to provide a balance to clinician and researcher assumptions, so the inclusion in both the predefined list and the extra strategies of many clinically not recommended strategies was intentional. Future research is needed to provide empirical evidence to inform decision-making about these approaches, for example, to identify when comfort eating is a healthier response compared to alternative behaviours, and when deliberate self-harm as an emotional self-regulation approach is a preferable alternative to an active suicide attempt.

This study has strengths and limitations. Strengths in relation to reporting guidelines^27^ include: a collaboration level of involvement from inception to completion, the focus on a genuine science outcome, reporting of platform use and public data availability, acknowledgement of contributors, and ethical approval being obtained. The impact on contributors and researchers will be explored in a future publication, and contributors will be informed of the findings.

Methodological limitations include the absence of training for public contributors, the anonymisation of contributors making further engagement in analysis challenging, and the need for more accessible pathways for contributors to be involved in analysis and in future citizen science projects. The ethnicity balance for contributors was close to population norms, but female contributors were over-represented, so approaches targeting male contributors are needed. Small participant numbers required that non-binary genders were not included and non-White British ethnicity sub-groups were combined in the regression modelling, so these sub-groups require further investigation. Further information on less well-known strategies, such as ASMR (Autonomous Sensory Meridian Response) may be useful. Finally, a more complete appraisal of the published literature on self-management strategies for various conditions and service settings could have further informed the development of the predefined strategies list.

This study has research, clinical and methodological implications. Further research is needed to understand self-management differences between people with lived experience who do versus do not use mental health services. Service-side barriers to providing self-management interventions include high staff turnover/shortage, lack of supervision and support for staff, and lack of senior clinical leadership^35^. Possible influences on individual-level decision-making about self-management include access to strategies such as prescribed medication, explanatory models and clinical advice, which may shape self-management choices, and the impact of anticipated discrimination and financial resources on social strategy usage. Whilst income did not predict use of the most frequently-used strategies (Supplementary material 3), future research might include consideration of disposable income (rather than income, as captured in this project), and other potential predictors of strategy use such as geographical location, to investigate influences on choice and impact of self-management strategies. Similarly, cross-cultural validation and refinement of the self-management strategies is warranted.

One clinical implication is the development of an ecologically valid list of *100 Ways to Support my Recovery* (Supplementary Material 4). This is intended to complement *100 Ways to Support Recovery*^36^, a guide to supporting recovery for mental health professionals which has been translated into 14 languages (www.researchintorecovery.com/100-ways-to-support-recovery-2nd-edition). Strategies that are not clinically recommended are included, because we contend that there may be value in making this information widely available. Evaluation is needed to establish the extent to which the clinical concern that including not recommended strategies will lead to emulative behaviour is offset by more positive impacts, e.g., the self-esteem enhancement from understanding that others use less recommended strategies, and having more awareness of options in preference to suicidal behaviour. Widespread availability, both directly online and from clinicians of *100 Ways to Support my Recovery* is indicated. Furthermore, the findings that both avoidance and non-clinically recommended strategies are frequently used have implications for clinical practice, e.g., the need for non-judgemental probing about an individual’s current approaches to living with their mental health issues. Particular attention should be given to identifying non-recommended strategies (e.g., non-prescribed drugs, overeating, ‘un-healthy’ sleep architecture and service avoidance) being currently used, in order to support decision-making about their use in the future.

One methodological implication in relation to future citizen science projects is that there is a need for a more mental health-specific platform, which is more designed for mental health projects. Our design was informed by the citizen science platform www.patientslikeme.com, on which individuals can share health-related experiences and coping strategies. However, our approach differs in using a transdiagnostic approach, because many people live with multiple long-term conditions and because we wanted to differentiate from biomedical statements on www.patientslikeme.com, such as ‘schizophrenia is a psychiatric disorder’. Specific issues needing consideration with existing platforms include the collection of informed consent and sociodemographic data about contributors^37,38^.

To our knowledge, this is the first transdiagnostic citizen mental health science project to be published in an academic journal. This project demonstrates the feasibility of involving large numbers of public contributors, and that the citizen science methodology has potential as a mental health-related knowledge creation approach, which extends traditional researcher-led science. Our finding that people use more avoidance, education and emotional self-regulation self-management strategies than previously known opens up new opportunities for evaluated innovation in supporting mental health recovery.

## Supplementary information

Supplementary Information

## Data Availability

De-identified data comprising individual participant data and a data dictionary will be available from the corresponding author (m.slade@nottingham.ac.uk) for research use with investigator support following publication for seven years.
