# Supplementary material for: Living with mental health issues: citizen science project on self-management strategies
Source: Npj Ment Health Res. 2025 Oct 11;4:50. doi: 10.1038/s44184-025-00166-2 (PMC12515248; doi:10.1038/s44184-025-00166-2)
Supplement: Supplementary file 1 — Supplementary Information [file 44184_2025_166_MOESM1_ESM.docx]

**Supplementary material**

| **Page** | **Contents** |
| --- | --- |
| 2 | Supplementary Material 1  Self-management questionnaire |
| 6 | Supplementary Material 2  Table S1: Frequency of use of predefined self-management strategies (N=77) |
| 9 | Supplementary Material 3  Usage predictors of most frequently used strategies (n=7) |
| 12 | Supplementary Material 4  100 ways to Support my Recovery |

**Supplementary Material 1**

**Self-management questionnaire**

**1. How do people manage their mental health challenges?**

**Who we are:**

We are a group of Mental Health Citizen Science researchers (including academics and public members) at the University of Nottingham, United Kingdom.

**What we are doing:**

Mental health is a state of mental well-being that enables people to cope with the stresses of life, realize their abilities, learn well, work well, and contribute to their community. We have worked with people who have experienced mental health challenges to create an initial list of strategies that they commonly use to manage their own mental health. We want to identify the ways individuals manage their mental health problems, by generating a long list of common mental health self-management strategies that people find helpful

**What we want you to do now:**

We want you to rate how commonly you use the mental health self-management strategies that people with lived experience of mental health problems and their carers have told us they use. We want you to tell us about other self-management strategies that you or others use in managing your mental health, that we have not enlisted.

**Who can participate:**

You must be 18 years or older, and resident in the United Kingdom.

**What will happen next:**

We will develop a long list of self-management strategies that people use to manage their mental health. We will analyse the most common strategies and share our findings through our website.

**Why you:**

We want to learn from the general public what works best in managing their mental health.

**Disclaimer:**

We know that even thinking about mental health challenges can feel difficult for some, so please do stop your participation if you find it distressing. In case of any distress, we have developed some resources on what you can do and organisations you can reach for help here: https://www.researchintorecovery.com/wp-content/uploads/2024/03/C-STACS-approved-distress-information-v1.0.pdf. However, we understand that everyone is different so you will need to work out what is helpful.

**How to access further information:** We’ve developed a detailed information sheet, for those people who want to know more about our study. You can access it in full here: https://www.researchintorecovery.com/wp-content/uploads/2024/03/C-STACS-Participant-Information-Sheet-Project-1-v1.0.pdf

**Consent and knowing more about our Citizen Scientists**

***This is to understand the characteristics of the citizen scientists participating in this project. Thank you for your time!***

**Please consent to the following, before you participate** (Tick all)

[ ] Your participation in rating mental health self-management strategies is voluntary

[ ] You confirmed that you are aged 18 years old and above.

[ ] You confirmed that you have read the Information Sheet (https://www.researchintorecovery.com/wp-content/uploads/2024/03/C-STACS-Participant-Information-Sheet-Project-1-v1.0.pdf) and understood it.

[ ] You confirm that you are a resident of the United Kingdom.

**What best describes you?** (A carer is anyone supporting someone with mental health problems in any capacity)

[ ] Carer to someone with a mental health problem.

[ ] Mental health service user.

[ ] Mental health worker.

[ ] Personal experience with mental health problems but never used services.

[ ] No personal experience of mental health problem.

**What gender to do you identify as?**

[ ] Female

[ ] Male

[ ] Non-binary

[ ] Prefer not to say

**What is your level of education?**

[ ] Early Years

[ ] Primary

[ ] Secondary

[ ] Degree level

[ ] Higher degree education

[ ] Prefer not to say

**How would you describe your ethnicity?**

[ ] Arab

[ ] Asian/Asian British

[ ] Black/Black British

[ ] Black African

[ ] Black Caribbean

[ ] Latin

[ ] Mixed Background

[ ] Gypsy, Roma or Irish traveller

[ ] White British

[ ] European

[ ] Not Mentioned

[ ] Prefer not to say

[ ] Not known

**What is your age category?**

[ ] 18-24

[ ] 25-34

[ ] 35-44

[ ] 45-54

[ ] 55-64

[ ] 65 and over

**Which of the following best describes your personal income?**

[ ] Less than £10,000

[ ] £10,000 - £25,000

[ ] £25,000 - £49,999

[ ] £50,000 - £74,999

[ ] £75,000 - £99,999

[ ] £100,000 or more

[ ] Prefer not to say

**Rating the self-management strategies you use (You have a total of 27 Questions to Answer)**

**Always** - This is the strategy that you use almost every day

**Very Often** - This is the strategy that you use moderately regularly for instance 3 - 4 days a week

**Sometimes** - This is the strategy that you use occasionally, rather than all the time

**Rarely** - This is the strategy that you use on rare occasions or you hardly use it

**Never** - This is the strategy that you never use or heard about

**I use these strategies to support my mental health**

1. Go to my GP (family doctor) to ask for support
2. I choose to take my medication as prescribed
3. Contact the local mental health crisis line for support
4. Go to my Recovery College
5. Engage with an online peer support group
6. Engage with a local in-person peer support group
7. Asking for Talking therapy
8. Spending time with peers with lived experience of mental health
9. Asking for cognitive behavioural therapy
10. Seek support from a mental health charity, e.g. Rethink, Samaritans
11. Ask my clinician for a specific biological treatment, e.g. medication
12. Organise (either through the NHS or privately) a talking therapy, e.g. counselling
13. Meditation
14. Mindfulness
15. Listening to music
16. I engage in exercise, e.g. walk/jog/run/ cycle
17. I spend time with nature, e.g., in parks, with animals
18. I engage in gardening
19. Mapping and writing a Wellness Recovery Action Plan (WRAP)
20. Use of the Wheel of Life to create a life balance
21. Use of Flower of Life tool to focus
22. Play with a pet [Always; Very often; Sometimes; Rarely; Never]
23. Take part in Yoga e.g. Trauma-informed yoga
24. Dance to loud music
25. Take part in volunteering activities
26. Engage in Self Improvement activities
27. Practising self-compassion
28. Participate in physical activity
29. Hug a pillow or a toy
30. Engagement with Cultural activity e.g. taking part in one’s heritage activities, learning about your heritage
31. Listening to mental health podcasts on any platform
32. Tracking of mental health symptoms with a mobile application
33. Activities to focus/reset on nervous system e.g. stretching, colouring, gaming
34. Journalling
35. Going to sleep early or changing my sleep patterns
36. Doing household chores e.g. tidying up, washing clothes
37. Learning new skills, e.g. new language
38. Doing Autonomous Sensory Meridian Response (ASMR) activities e.g. talking or moving softly
39. Avoiding situations or people that trigger my distress
40. Participating in a reading group
41. Participating in a music group
42. Participating in an art group or watching art at the gallery
43. Participating in a craft group
44. Participating in a sports activity
45. Participating in activism
46. Enjoying quality family time
47. Participating in a holistic wellness activity
48. Being with Support systems e.g. trusted friends, families
49. Meet with friends
50. Have a cup of tea
51. Take a bath or shower
52. Cook or bake your favourite foods
53. Look at pictures of destinations you would like to visit
54. Use social media
55. Play Sudoku or other brain teasers
56. Taking time off work
57. Organising a social activity
58. Distracting myself
59. Counting my blessings
60. Having sex
61. Going to the club or a pub
62. Going gambling
63. Going to an art gallery
64. Watching TV
65. Getting drunk
66. Taking illicit drugs e.g. cocaine
67. Keeping busy at work
68. Reading
69. Smoking cigarettes
70. Practice strategies learned in therapy
71. Watching a sports match e.g. football, Rugby
72. Going to the firepit
73. Daydreaming
74. Bird-watching
75. Being with others in my place of worship, e.g. church, synagogue, mosque, temple
76. Engaging in faith-based practice e.g. praying, Singing and reciting religious books
77. Listening to spiritual leaders

**Can you tell us any mental health self-management strategies you use which we haven’t included?**

[Free text]

**Supplementary Material 2**

**Table S1: Frequency of use of predefined self-management strategies (N=77)**

|  | **Frequency**  *Never (0) to Always (4)*  Median (IQR) |  |  |  | **Difference**  Unadjusted p-value | **Difference**  p-value with Bonferroni correction | **Difference**  p-value with False Discovery Rate adjustment |
| --- | --- | --- | --- | --- | --- | --- | --- |
| **Contributors** | **All** | **No lived experience** | **Lived experience, no service use** | **Mental health service user** |  |  |  |
| *n* | *1,116* | *151* | *497* | *468* |  |  |  |
| **Accessing support from the mental health system** (Aggregated use of 12 strategies) | **0 (0,1)** | **0 (0,0)** | **0 (0,0)** | **0 (0,2)** | **<0·01** | **<0.01** | **<0.01** |
| 1. I choose to take my medication as prescribed | 3 (0,4) | 0 (0,4) | 1 (0,4) | 4 (1,4) | <0·01 | <0.01 | <0.01 |
| 1. Go to my GP (family doctor) to ask for support | 1 (0,2) | 1 (0,2) | 1 (0,1) | 1 (1,2) | <0·01 | <0.01 | <0.01 |
| 1. Asking for talking therapy | 1 (0,2) | 0 (0,1) | 0 (0,1) | 2 (1,3) | <0·01 | <0.01 | <0.01 |
| 1. Spending time with peers with lived experience of mental health | 1 (0,2) | 0 (0,2) | 0 (0,2) | 1 (0,2) | <0·01 | <0.01 | <0.01 |
| 1. Organise (either through the NHS or privately) a talking therapy, e.g. counselling | 1 (0,2) | 0 (0,1) | 0 (0,2) | 2 (1,3) | <0·01 | <0.01 | <0.01 |
| 1. Ask my clinician for a specific biological treatment, e.g. medication | 0 (0,2) | 0 (0,0) | 0 (0,1) | 1 (0,2) | <0·01 | <0.01 | <0.01 |
| 1. Contact my local mental health crisis line for support | 0 (0,1) | 0 (0,0) | 0 (0,0) | 0 (0,1) | <0·01 | <0.01 | <0.01 |
| 1. Engage with an online peer support group | 0 (0,1) | 0 (0,0) | 0 (0,0) | 0 (0,1) | <0·01 | <0.01 | <0.01 |
| 1. Asking for cognitive behavioural therapy | 0 (0,1) | 0 (0,0) | 0 (0,1) | 1 (0,2) | <0·01 | <0.01 | <0.01 |
| 1. Seek support from a mental health charity e.g. Rethink, Samaritans | 0 (0,1) | 0 (0,0) | 0 (0,0) | 1 (0,2) | <0·01 | <0.01 | <0.01 |
| 1. Go to my Recovery College | 0 (0,0) | 0 (0,0) | 0 (0,0) | 0 (0,0) | <0·01 | <0.01 | <0.01 |
| 1. Engage with a local in-person peer support group | 0 (0,0) | 0 (0,0) | 0 (0,0) | 0 (0,1) | <0·01 | <0.01 | <0.01 |
| **Individual approaches (clinically recommended)** (Aggregated use of 44 strategies) | **1 (0,2)** | **1 (0,2)** | **1 (0,2)** | **1 (0,2)** | **<0·01** | **<0.01** | **<0.01** |
| 1. Listening to music | 3 (2,4) | 3 (2,4) | 3 (2,4) | 3 (2,4) | 0·81 | 1.00 | 0.83 |
| 1. Watching TV | 3 (2,4) | 3 (2,3) | 3 (2,4) | 3 (2,4) | 0·02 | 1.00 | 0.04 |
| 1. Distracting myself | 3 (2,4) | 2 (1,3) | 3 (2,4) | 3 (2,4) | <0·01 | 0.45 | 0.01 |
| 1. Avoiding situations or people that trigger my distress | 3 (2,3·25) | 2 (1,3) | 3 (2,3) | 3 (2,4) | <0·01 | <0.01 | <0.01 |
| 1. Have a cup of tea | 3 (1,4) | 3 (0,4) | 2 (1,4) | 3 (1,4) | 0·17 | 1.00 | 0.22 |
| 1. Spending time in nature, e.g. in parks, with animals | 2 (2,3) | 3 (1,3) | 2 (1·25,4) | 2 (2,3) | 0·83 | 1.00 | 0.84 |
| 1. Taking a bath or shower | 2 (2,3) | 3 (2,4) | 2 (2,3) | 2 (1,3) | 0·02 | 1.00 | 0.04 |
| 1. Exercising, e.g. walk/jog/run/cycle | 2 (1,4) | 3 (2,4) | 2·5 (1,4) | 2 (1,3) | 0·03 | 1.00 | 0.05 |
| 1. Playing with a pet | 2 (0,4) | 2 (0,4) | 2 (0,4) | 2 (0,4) | 0·51 | 1.00 | 0.58 |
| 1. Physical activity | 2 (1,3) | 2 (1,4) | 2 (1,3) | 2 (1,3) | 0·05 | 1.00 | 0.09 |
| 1. Going to sleep early or changing my sleep patterns | 2 (1,3) | 2 (0,2) | 2 (1,3) | 2 (1,3) | 0·01 | 1.00 | 0.03 |
| 1. Doing household chores, e.g. tidying up, washing clothes | 2 (1,3) | 2 (1,3) | 2 (1,3) | 2 (1,3) | 0·69 | 1.00 | 0.75 |
| 1. Daydreaming | 2 (1,3) | 2 (1,3) | 2 (1,3) | 2 (1,3) | 0·26 | 1.00 | 0.31 |
| 1. Reading | 2 (1,3) | 2 (1,3) | 2 (1,3) | 2 (1,3) | 0·77 | 1.00 | 0.80 |
| 1. Mindfulness | 2 (0,3) | 2 (0,3) | 2 (0,3) | 2 (0,3) | 0·04 | 1.00 | 0.07 |
| 1. Activities to focus/reset on nervous system, e.g. stretching, colouring, gaming | 2 (0,3) | 1 (0,2) | 2 (0,3) | 2 (1,3) | <0·01 | 0.01 | <0.01 |
| 1. Counting my blessings | 2 (0,3) | 2 (0,3) | 2 (0,3) | 2 (0,3) | 0·36 | 1.00 | 0.42 |
| 1. Keeping busy at work | 2 (0,3) | 2 (0,3) | 2 (0,3) | 2 (0,3) | 0·18 | 1.00 | 0.23 |
| 1. Cooking or baking my favourite foods | 2 (0,2) | 2 (0·5,3) | 2 (0,2) | 2 (0,2) | <0·01 | 0.32 | 0.01 |
| 1. Hugging a pillow or a toy | 1 (0,3) | 1 (0,2) | 1 (0,3) | 2 (0,3) | 0·16 | 1.00 | 0.22 |
| 1. Playing Sudoku or other brain teasers | 1 (0,3) | 1 (0,3) | 1 (0,3) | 2 (0,3) | <0·01 | 0.12 | <0.01 |
| 1. Meditation | 1 (0,2) | 0 (0,2) | 1 (0,2) | 1 (0,3) | <0·01 | <0.01 | <0.01 |
| 1. Gardening | 1 (0,2) | 2 (0,3) | 1 (0,2) | 1 (0,2) | 0·11 | 1.00 | 0.16 |
| 1. Dancing to loud music | 1 (0,2) | 1 (0,2) | 1 (0,2) | 1 (0,2) | 0·16 | 1.00 | 0.22 |
| 1. Taking part in volunteering activities | 1 (0,2) | 1 (0,2) | 0 (0,2) | 1 (0,2) | <0·01 | 0.16 | <0.01 |
| 1. Self improvement activities | 1 (0,2) | 1 (0,3) | 1 (0,2) | 2 (0,2) | <0·01 | 0.69 | 0.02 |
| 1. Practising self-compassion | 1 (0,2) | 1 (0,2·5) | 1 (0,2) | 2 (0,2) | <0·01 | 0.15 | <0.01 |
| 1. Learning new skills, e.g. new language | 1 (0,2) | 1 (0,2) | 1 (0,2) | 1 (0,2) | 0·10 | 1.00 | 0.15 |
| 1. Looking at pictures of destinations you would like to visit | 1 (0,2) | 2 (0,2·5) | 1 (0,2) | 1 (0,2) | 0·01 | 0.77 | 0.02 |
| 1. Taking time off work | 1 (0,2) | 1 (0,2) | 1 (0,2) | 1 (0,2) | <0·01 | <0.01 | <0.01 |
| 1. Practising strategies learned in therapy | 1 (0,2) | 0 (0,2) | 0 (0,2) | 2 (1,3) | <0·01 | <0.01 | <0.01 |
| 1. Engagement with cultural activity, e.g. taking part in one’s heritage activities, learning about your heritage | 0 (0,2) | 1 (0,2) | 0 (0,2) | 0 (0,2) | 0·11 | 1.00 | 0.15 |
| 1. Listening to mental health podcasts | 0 (0,2) | 0 (0,1) | 0 (0,1·75) | 1 (0,2) | <0·01 | 0.25 | <0.01 |
| 1. Journalling | 0 (0,2) | 0 (0,1) | 0 (0,2) | 1 (0,2) | <0·01 | <0.01 | <0.01 |
| 1. Participating in a holistic wellness activity | 0 (0,2) | 0 (0,1·5) | 0 (0,2) | 0 (0,2) | 0·17 | 1.00 | 0.22 |
| 1. Bird-watching | 0 (0,2) | 0 (0,2) | 0 (0,2) | 0 (0,2) | 0·73 | 1.00 | 0.77 |
| 1. Yoga, e.g. Trauma-informed yoga | 0 (0,1) | 0 (0,0) | 0 (0,1) | 0 (0,1) | 0·70 | 1.00 | 0.75 |
| 1. Tracking of mental health symptoms with a mobile application | 0 (0,1) | 0 (0,0) | 0 (0,1) | 0 (0,1) | <0·01 | 0.16 | <0.01 |
| 1. Going to an art gallery | 0 (0,1) | 0 (0,1) | 0 (0,2) | 0 (0,1) | 0·51 | 1.00 | 0.58 |
| 1. Mapping and writing a Wellness Recovery Action Plan (WRAP) | 0 (0,0) | 0 (0,0) | 0 (0,0) | 0 (0,1) | <0·01 | 0.01 | <0.01 |
| 1. Use of the Wheel of Life to create a life balance | 0 (0,0) | 0 (0,0) | 0 (0,0) | 0 (0,0) | 0·89 | 1.00 | 0.89 |
| 1. Use of Flower of Life tool to focus | 0 (0,0) | 0 (0,0) | 0 (0,0) | 0 (0,0) | 0·04 | 1.00 | 0.08 |
| 1. Doing Autonomous Sensory Meridian Response (ASMR) activities, e.g. talking, moving softly | 0 (0,0) | 0 (0,0) | 0 (0,0) | 0 (0,0) | 0·26 | 1.00 | 0.31 |
| 1. Using a firepit | 0 (0,0) | 0 (0,0) | 0 (0,0) | 0 (0,0) | 0·24 | 1.00 | 0.30 |
| **Individual approaches (clinically not recommended)**  (Aggregated use of 4 strategies) | **0 (0,2)** | **0 (0,2)** | **0 (0,2)** | **0 (0,2)** | **0.14** | **0.55** | **0.14** |
| 1. Getting drunk | 0 (0,1) | 0 (0,1) | 0 (0,1) | 0 (0,1) | 0·13 | 1.00 | 0.19 |
| 1. Gambling | 0 (0,0) | 0 (0,0) | 0 (0,0) | 0 (0,0) | 0·54 | 1.00 | 0.60 |
| 1. Taking illicit drugs, e.g. cocaine | 0 (0,0) | 0 (0,0) | 0 (0,0) | 0 (0,0) | 0·18 | 1.00 | 0.23 |
| 1. Smoking cigarettes | 0 (0,0) | 0 (0,0) | 0 (0,0) | 0 (0,0) | 0·05 | 1.00 | 0.08 |
| **Social approaches**  (Aggregated use of 14 strategies) | **1 (0,3)** | **2 (0,3)** | **1 (0,2)** | **1 (0,3)** | **<0·01** | **<0.01** | **<0.01** |
| 1. Using social media | 3 (2,4) | 3 (2,4) | 2 (1,3) | 3 (2,4) | <0·01 | 0.68 | 0.02 |
| 1. Enjoying quality family time | 2 (1,3) | 3 (2,4) | 2 (1,3) | 2 (1,3) | <0·01 | 0.45 | 0.01 |
| 1. Being with support systems, e.g. trusted friends, families | 2 (1,3) | 2 (1,3) | 2 (1,3) | 2 (2,3) | 0·05 | 1.00 | 0.08 |
| 1. Meeting with friends | 2 (1,3) | 2 (1,4) | 2 (1,3) | 2 (1,3) | <0·01 | 0.31 | 0.01 |
| 1. Organising a social activity | 1 (0,2) | 1 (0,2) | 1 (0,2) | 1 (0,2) | 0·11 | 1.00 | 0.15 |
| 1. Participating in a sports activity | 0 (0,2) | 1 (0,3) | 0 (0,2) | 0 (0,1) | <0·01 | <0.01 | <0.01 |
| 1. Going to a club or pub | 0 (0,1·25) | 0 (0,2) | 0 (0,2) | 0 (0,1) | 0·46 | 1.00 | 0.54 |
| 1. Participating in an art group | 0 (0,1) | 0 (0,1) | 0 (0,0) | 0 (0,1) | 0·10 | 1.00 | 0.15 |
| 1. Participating in a craft group | 0 (0,1) | 0 (0,1) | 0 (0,0) | 0 (0,1) | <0·01 | 0.07 | <0.01 |
| 1. Participating in activism | 0 (0,1) | 0 (0,0) | 0 (0,1) | 0 (0,1) | <0·01 | 0.02 | <0.01 |
| 1. Having sex | 0 (0,1) | 0 (0,2) | 0 (0,1) | 0 (0,1) | 0·02 | 1.00 | 0.04 |
| 1. Watching a sports match e.g. football, rugby | 0 (0,1) | 0 (0,2) | 0 (0,1) | 0 (0,1) | 0·02 | 1.00 | 0.04 |
| 1. Participating in a reading group | 0 (0,0) | 0 (0,0) | 0 (0,0) | 0 (0,0) | 0·58 | 1.00 | 0.64 |
| 1. Participating in a music group | 0 (0,0) | 0 (0,0) | 0 (0,0) | 0 (0,0) | 0·04 | 1.00 | 0.08 |
| **Spiritual engagement**  (Aggregated use of 3 strategies) | **0 (0,0)** | **0 (0,1)** | **0 (0,0)** | **0 (0,1)** | **<0·01** | **<0.01** | **<0.01** |
| 1. Being with others in my place of worship, e.g. church, synagogue, mosque, temple | 0 (0,0) | 0 (0,1) | 0 (0,0) | 0 (0,1) | 0·09 | 1.00 | 0.14 |
| 1. Engaging in faith-based practice, e.g. praying, singing, reciting religious books | 0 (0,0) | 0 (0,1) | 0 (0,0) | 0 (0,1) | 0·03 | 1.00 | 0.05 |
| 1. Listening to spiritual leaders | 0 (0,0) | 0 (0,0) | 0 (0,0) | 0 (0,1) | 0·11 | 1.00 | 0.15 |

**Supplementary Material 3**

**Usage predictors of most frequently used strategies (n=7)**

**Table S3.1 Predictors of usage of ‘I choose to take my medication as prescribed’**

| **Predictor** | **Odds Ratio** (95%CI) | **p-value** |
| --- | --- | --- |
| Experience group: Lived experience no service use | 1 (0.66 to 1.51) | 1 |
| Experience group: Mental health service user | 3.39 (2.22 to 5.18) | <0.01 |
| Gender: Female | 1.24 (0.90 to 1.72) | 0.19 |
| Education: Degree/Higher degree | 0.67 (0.49 to 0.90) | <0.01 |
| Ethnicity: Other | 0.82 (0.56 to 1.19) | 0.29 |
| Age: 25-34 | 1.6 (0.87 to 2.97) | 0.13 |
| Age: 35-44 | 1.91 (1.08 to 3.38) | 0.03 |
| Age 45-54 | 2.64 (1.53 to 4.54) | <0.01 |
| Age: 55-64 | 2.21 (1.32 to 3.68) | <0.01 |
| Age: 65+ | 2.04 (1.07 to 3.89) | 0.03 |
| Income: £10,000-£24,999 | 1.22 (0.87 to 1.71) | 0.25 |
| Income: £24,000-£49,999 | 0.95 (0.64 to 1.39) | 0.77 |
| Income: £50,000-£74,999 | 0.68 (0.39 to 1.18) | 0.17 |
| Income: £75,000-£99,999 | 0.53 (0.23 to 1.22) | 0.13 |
| Income: £100,000+ | 0.61 (0.24 to 1.52) | 0.29 |

**Table S3.2 Predictors of usage of ‘Listening to music’**

| **Predictor** | **Odds Ratio** (95%CI) | **p-value** |
| --- | --- | --- |
| Experience group: Lived experience no service use | 0.93 (0.63 to 1.37) | 0.71 |
| Experience group: Mental health service user | 0.8 (0.54 to 1.19) | 0.28 |
| Gender: Female | 1.05 (0.77 to 1.44) | 0.76 |
| Education: Degree/Higher degree | 0.99 (0.74 to 1.32) | 0.95 |
| Ethnicity: Other | 1.22 (0.85 to 1.73) | 0.28 |
| Age: 25-34 | 0.38 (0.20 to 0.71) | <0.01 |
| Age: 35-44 | 0.22 (0.12 to 0.40) | <0.01 |
| Age 45-54 | 0.26 (0.15 to 0.46) | <0.01 |
| Age: 55-64 | 0.2 (0.12 to 0.34) | <0.01 |
| Age: 65+ | 0.13 (0.07 to 0.26) | <0.01 |
| Income: £10,000-£24,999 | 1.19 (0.85 to 1.65) | 0.31 |
| Income: £24,000-£49,999 | 1.19 (0.82 to 1.71) | 0.37 |
| Income: £50,000-£74,999 | 0.67 (0.40 to 1.14) | 0.14 |
| Income: £75,000-£99,999 | 0.68 (0.32 to 1.43) | 0.31 |
| Income: £100,000+ | 1.06 (0.46 to 2.46) | 0.89 |

**Table S3.3 Predictors of usage of ‘Watching TV’**

| **Predictor** | **Odds Ratio** (95%CI) | **p-value** |
| --- | --- | --- |
| Experience group: Lived experience no service use | 0.95 (0.64 to 1.39) | 0.78 |
| Experience group: Mental health service user | 1.2 (0.80 to 1.79) | 0.37 |
| Gender: Female | 1.55 (1.14 to 2.11) | <0.01 |
| Education: Degree/Higher degree | 0.66 (0.50 to 0.88) | <0.01 |
| Ethnicity: Other | 0.66 (0.46 to 0.94) | 0.02 |
| Age: 25-34 | 0.93 (0.50 to 1.74) | 0.83 |
| Age: 35-44 | 1.04 (0.58 to 1.84) | 0.91 |
| Age 45-54 | 0.91 (0.53 to 1.56) | 0.72 |
| Age: 55-64 | 1.1 (0.65 to 1.83) | 0.73 |
| Age: 65+ | 0.85 (0.46 to 1.57) | 0.6 |
| Income: £10,000-£24,999 | 1.24 (0.89 to 1.72) | 0.2 |
| Income: £24,000-£49,999 | 1 (0.69 to 1.45) | 0.99 |
| Income: £50,000-£74,999 | 0.91 (0.55 to 1.51) | 0.72 |
| Income: £75,000-£99,999 | 0.79 (0.38 to 1.64) | 0.53 |
| Income: £100,000+ | 1.04 (0.41 to 2.63) | 0.93 |

**Table S3.4 Predictors of usage of ‘Distracting myself’**

| **Predictor** | **Odds Ratio** (95%CI) | **p-value** |
| --- | --- | --- |
| Experience group: Lived experience no service use | 1.55 (1.03 to 2.32) | 0.03 |
| Experience group: Mental health service user | 1.66 (1.09 to 2.53) | 0.02 |
| Gender: Female | 1.72 (1.26 to 2.36) | <0.01 |
| Education: Degree/Higher degree | 1.2 (0.90 to 1.60) | 0.21 |
| Ethnicity: Other | 0.88 (0.61 to 1.25) | 0.47 |
| Age: 25-34 | 0.98 (0.52 to 1.84) | 0.95 |
| Age: 35-44 | 0.62 (0.35 to 1.10) | 0.1 |
| Age 45-54 | 0.74 (0.42 to 1.28) | 0.28 |
| Age: 55-64 | 0.51 (0.30 to 0.86) | 0.01 |
| Age: 65+ | 0.56 (0.30 to 1.06) | 0.07 |
| Income: £10,000-£24,999 | 0.93 (0.67 to 1.29) | 0.66 |
| Income: £24,000-£49,999 | 0.79 (0.54 to 1.14) | 0.2 |
| Income: £50,000-£74,999 | 0.71 (0.42 to 1.21) | 0.21 |
| Income: £75,000-£99,999 | 1.12 (0.54 to 2.32) | 0.75 |
| Income: £100,000+ | 1.52 (0.59 to 3.94) | 0.39 |

**Table S3.5 Predictors of usage of ‘Avoiding situations or people that trigger my distress’**

| **Predictor** | **Odds Ratio** (95%CI) | **p-value** |
| --- | --- | --- |
| Experience group: Lived experience no service use | 2.59 (1.73 to 3.86) | <0.01 |
| Experience group: Mental health service user | 3.89 (2.57 to 5.89) | <0.01 |
| Gender: Female | 1.35 (0.99 to 1.84) | 0.06 |
| Education: Degree/Higher degree | 1.33 (0.99 to 1.77) | 0.05 |
| Ethnicity: Other | 1.05 (0.73 to 1.49) | 0.8 |
| Age: 25-34 | 2.56 (1.38 to 4.73) | <0.01 |
| Age: 35-44 | 2.26 (1.28 to 3.97) | <0.01 |
| Age 45-54 | 2.39 (1.39 to 4.10) | <0.01 |
| Age: 55-64 | 2.23 (1.34 to 3.71) | <0.01 |
| Age: 65+ | 1.59 (0.84 to 2.99) | 0.15 |
| Income: £10,000-£24,999 | 0.89 (0.64 to 1.23) | 0.48 |
| Income: £24,000-£49,999 | 0.62 (0.43 to 0.90) | 0.01 |
| Income: £50,000-£74,999 | 0.46 (0.27 to 0.78) | <0.01 |
| Income: £75,000-£99,999 | 0.54 (0.26 to 1.11) | 0.09 |
| Income: £100,000+ | 0.77 (0.30 to 1.94) | 0.57 |

**Table S3.6 Predictors of usage of ‘Have a cup of tea’**

| **Predictor** | **Odds Ratio** (95%CI) | **p-value** |
| --- | --- | --- |
| Experience group: Lived experience no service use | 0.7 (0.47 to 1.04) | 0.08 |
| Experience group: Mental health service user | 0.71 (0.48 to 1.07) | 0.1 |
| Gender: Female | 1.54 (1.13 to 2.10) | <0.01 |
| Education: Degree/Higher degree | 1.19 (0.89 to 1.58) | 0.24 |
| Ethnicity: Other | 0.93 (0.65 to 1.32) | 0.67 |
| Age: 25-34 | 1.95 (1.08 to 3.51) | 0.03 |
| Age: 35-44 | 3.07 (1.77 to 5.30) | <0.01 |
| Age 45-54 | 4.26 (2.53 to 7.19) | <0.01 |
| Age: 55-64 | 3.98 (2.43 to 6.53) | <0.01 |
| Age: 65+ | 3.48 (1.87 to 6.48) | <0.01 |
| Income: £10,000-£24,999 | 0.99 (0.72 to 1.37) | 0.97 |
| Income: £24,000-£49,999 | 1.14 (0.79 to 1.64) | 0.5 |
| Income: £50,000-£74,999 | 0.73 (0.44 to 1.22) | 0.23 |
| Income: £75,000-£99,999 | 1.33 (0.64 to 2.75) | 0.44 |
| Income: £100,000+ | 0.69 (0.27 to 1.80) | 0.45 |

**Table S3.7 Predictors of usage of ‘Using social media’**

| **Predictor** | **Odds Ratio** (95%CI) | **p-value** |
| --- | --- | --- |
| Experience group: Lived experience no service use | 0.91 (0.62 to 1.33) | 0.63 |
| Experience group: Mental health service user | 1.61 (1.08 to 2.39) | 0.02 |
| Gender: Female | 1.42 (1.04 to 1.94) | 0.03 |
| Education: Degree/Higher degree | 0.62 (0.47 to 0.83) | <0.01 |
| Ethnicity: Other | 1.18 (0.82 to 1.69) | 0.38 |
| Age: 25-34 | 0.34 (0.18 to 0.64) | <0.01 |
| Age: 35-44 | 0.38 (0.22 to 0.67) | <0.01 |
| Age 45-54 | 0.34 (0.20 to 0.58) | <0.01 |
| Age: 55-64 | 0.41 (0.25 to 0.69) | <0.01 |
| Age: 65+ | 0.44 (0.24 to 0.83) | 0.01 |
| Income: £10,000-£24,999 | 1.14 (0.83 to 1.57) | 0.42 |
| Income: £24,000-£49,999 | 1.05 (0.73 to 1.51) | 0.78 |
| Income: £50,000-£74,999 | 0.69 (0.41 to 1.15) | 0.15 |
| Income: £75,000-£99,999 | 0.63 (0.30 to 1.29) | 0.21 |
| Income: £100,000+ | 1.01 (0.39 to 2.59) | 0.99 |

**Supplementary Material 4**

**Integrated list of strategies**

*100 Ways to Support my Recovery*

This is a list of strategies that people use to cope with, manage and live with mental health problems. The list was created by merging three sources: published research, Patient and Public Involvement suggestions and a citizen science project on self-management involving 1,116 public contributors. Some of the strategies are not recommended by mental health professionals, but are included because they are used by some people.

| **Support from the mental health system** |
| --- |
| 1. Going to my GP (family doctor) to ask for support |
| 1. Seeking support from a mental health charity e.g. Rethink, Samaritans |
| 1. Contacting my local mental health crisis line for support |
| 1. Asking the NHS for a talking therapy, e.g. cognitive behavioural therapy |
| 1. Organising a talking therapy privately, e.g. counselling |
| 1. Asking for a specific biological treatment, e.g. medication, ECT |
| 1. Choosing to take my medication as prescribed |
| 1. Going to my Recovery College |
| 1. Spending time with supportive peers with lived experience of mental health |
| 1. Engaging with an online or local in-person peer support group |
| **Things you can do by yourself which would be recommended by a mental health professional** |
| 1. Listening to music |
| 1. Dancing to loud music |
| 1. Watching TV or listening to radio or streaming a film |
| 1. Distracting myself, e.g. jigsaws, playing games on my mobile, building Lego |
| 1. Hugging a pillow or a toy |
| 1. Crying, e.g. crying myself to sleep, screaming into the void |
| 1. Laughing, e.g. humour, telling jokes, watching comedy live or on TV |
| 1. Gratitude, e.g. practising a grateful attitude, counting my blessings, seeing my family thrive |
| 1. Mindfulness, e.g. being aware and in the moment, without judgement |
| 1. Meditation, e.g. breathing techniques. |
| 1. Positive thinking, e.g. positive affirmations |
| 1. Acceptance and resilience, e.g. accepting myself, knowing that I’ve felt bad before and it will pass, remembering that low mood and stress are natural occurrences in life |
| 1. Practising self-compassion, e.g. having a do-nothing day |
| 1. Daydreaming |
| 1. Yoga, e.g. Trauma-informed yoga |
| 1. Participating in a holistic wellness activity |
| 1. Resetting the nervous system, e.g. stretching, colouring, gaming, worry worms, tapping, fidget toys, stimming, cold showers, vagus nerve stimulation |
| 1. Doing ASMR (Autonomous Sensory Meridian Response) activities, e.g. talking, moving softly |
| 1. Emotional self-regulation, e.g. Delta wave music, looking through photos of happy times, standing barefoot on wet grass, Shaolin practice |
| 1. Playing Sudoku or other brain teasers |
| 1. Reading books, listening to audiobooks, writing a family memoir |
| 1. Leisure, e.g. playing games like chess, relaxing, hobbies like plane-spotting, collecting, people-watching, making jam, cloud-watching |
| 1. Self care, e.g. have a cup of tea, take a bath or shower, shopping, getting a massage, using a weighted blanket, cutting my hair, ordering food so I don’t have to cook |
| 1. Exercising, e.g. walk/jog/run/cycle |
| 1. Doing household chores, e.g. tidying up, washing clothes |
| 1. Improving my living space, e.g. rearranging things, decluttering, decorating |
| 1. Healthy eating |
| 1. Cooking or baking my favourite foods |
| 1. Avoid alcohol, e.g. stop or cut down drinking, keep drinking to the weekend |
| 1. Spending time in nature, e.g. in parks, with animals, walking along a beach |
| 1. Gardening |
| 1. Using a firepit |
| 1. Playing with a pet, e.g. talking to my dog about my problems |
| 1. Engaging with animals and nature, e.g. bird-watching, butterfly-spotting, forest bathing, open water swimming, tree-planting |
| 1. Looking at pictures of destinations you would like to visit |
| 1. Travelling, e.g. going on holiday, going for a drive, doing something spontaneous |
| 1. Going to an art gallery, cinema, theatre or festival |
| 1. Engaging with cultural activities, e.g. taking part in or learning about one’s heritage, learning about other cultures |
| 1. Self improvement activities |
| 1. Learning new skills, e.g. new language |
| 1. Learning about mental health, e.g. self-help books, stress management courses, understanding the impact of traumas |
| 1. Listening to mental health podcasts |
| 1. Adult education, especially about non-mental health topics, e.g. internet research, short courses, genealogy |
| 1. Making lists and planning for the future, e.g. daily task list, sorting finances, setting alarms to remember to eat and drink |
| 1. Going to sleep early or changing my sleep patterns |
| 1. Keeping busy in work, e.g. increasing time in paid work |
| 1. Taking time off work, e.g. reducing time in paid work, talking to manager |
| 1. Keeping busy in volunteering activities |
| 1. Reducing time spent in volunteering activities |
| 1. Using mental health apps, e.g. Calm, Stay Alive, Loona, Wysa, Happify, Aura, Headspace |
| 1. Tracking of mental health symptoms with a mobile app |
| 1. Journalling |
| 1. Using the Wheel of Life to create a life balance |
| 1. Using the Flower of Life tool to focus |
| 1. Using a Wellness Recovery Action Plan (WRAP) |
| 1. Practising strategies learned in therapy |
| 1. Avoiding situations or people that trigger my distress |
| 1. Avoiding social media or devices, e.g. social media timeouts, block specific people, don’t answer emails, turn off / reduce device usage |
| **Things you can do by yourself which would NOT be recommended by a mental health professional** |
| 1. Avoiding thinking about my problems, e.g. compartmentalising or dissociating from my difficulties |
| 1. Complaining and arguing |
| 1. Isolating myself, e.g. staying in bed, lying in bed, keeping to myself, being alone, disappearing, avoiding romantic relationships |
| 1. Getting drunk |
| 1. Gambling |
| 1. Taking illegal drugs, e.g. cocaine |
| 1. Taking non-prescribed medications, e.g. weed/cannabis, psilocybin, sleeping tablets, un-prescribed antidepressants |
| 1. Smoking cigarettes |
| 1. Using complementary therapies |
| 1. Overeating |
| 1. Sleeping less, e.g. not sleeping |
| 1. Sleeping more, e.g. napping, sleeping for a long time |
| 1. Avoiding mental health services, e.g. specific workers or services |
| 1. Deliberate self-harm, e.g. cutting myself, planning to end my life, starving myself, taking risks |
| **Things you can do with others** |
| 1. Using social media |
| 1. Helping others |
| 1. Enjoying quality family time |
| 1. Being with support systems, e.g. trusted friends |
| 1. Socialising, e.g. meeting with other people |
| 1. Organising a social activity |
| 1. Participating in a sports activity |
| 1. Going to a club or pub with other people |
| 1. Participating in an art group, e.g. scrap-booking, textiles |
| 1. Participating in a craft group, e.g. knitting, writing, poetry, wood-carving, crochet |
| 1. Participating in activism |
| 1. Participating in a reading / writing group, e.g. fan fiction |
| 1. Participating in a music group, e.g. choir, singing group, orchestra |
| 1. Having sex, e.g. masturbation alone, with others |
| 1. Watching a sports match e.g. football, rugby |
| **Spiritual approaches you can use** |
| 1. Being with others in my place of worship, e.g. church, mosque, Pagan festival, synagogue, temple |
| 1. Engaging in faith-based practice, e.g. praying, singing, reading / reciting religious books, chanting, listening to spiritual podcasts, interfaith activities |
| 1. Listening to spiritual leaders, in-person or online |
